# Identification of Patients in Need of Advanced Care for Depression Using Data Extracted From a Statewide Health Information Exchange: A Machine Learning Approach

**DOI:** 10.2196/13809

**Published:** 2019-07-22

**Authors:** Suranga N Kasthurirathne, Paul G Biondich, Shaun J Grannis, Saptarshi Purkayastha, Joshua R Vest, Josette F Jones

**Affiliations:** 1 Center for Biomedical Informatics Regenstrief Institute Indianapolis, IN United States; 2 Indiana University Fairbanks School of Public Health Indianapolis, IN United States; 3 Indiana University School of Medicine Indianapolis, IN United States; 4 Indiana University School of Informatics and Computing Indianapolis, IN United States

**Keywords:** depression, supervised machine learning, delivery of health care

## Abstract

**Background:**

As the most commonly occurring form of mental illness worldwide, depression poses significant health and economic burdens to both the individual and community. Different types of depression pose different levels of risk. Individuals who suffer from mild forms of depression may recover without any assistance or be effectively managed by primary care or family practitioners. However, other forms of depression are far more severe and require advanced care by certified mental health providers. However, identifying cases of depression that require advanced care may be challenging to primary care providers and health care team members whose skill sets run broad rather than deep.

**Objective:**

This study aimed to leverage a comprehensive range of patient-level diagnostic, behavioral, and demographic data, as well as past visit history data from a statewide health information exchange to build decision models capable of predicting the need of advanced care for depression across patients presenting at Eskenazi Health, the public safety net health system for Marion County, Indianapolis, Indiana.

**Methods:**

Patient-level diagnostic, behavioral, demographic, and past visit history data extracted from structured datasets were merged with outcome variables extracted from unstructured free-text datasets and were used to train random forest decision models that predicted the need of advanced care for depression across (1) the overall patient population and (2) various subsets of patients at higher risk for depression-related adverse events; patients with a past diagnosis of depression; patients with a Charlson comorbidity index of ≥1; patients with a Charlson comorbidity index of ≥2; and all unique patients identified across the 3 above-mentioned high-risk groups.

**Results:**

The overall patient population consisted of 84,317 adult (aged ≥18 years) patients. A total of 6992 (8.29%) of these patients were in need of advanced care for depression. Decision models for high-risk patient groups yielded area under the curve (AUC) scores between 86.31% and 94.43%. The decision model for the overall patient population yielded a comparatively lower AUC score of 78.87%. The variance of optimal sensitivity and specificity for all decision models, as identified using Youden J Index, is as follows: sensitivity=68.79% to 83.91% and specificity=76.03% to 92.18%.

**Conclusions:**

This study demonstrates the ability to automate screening for patients in need of advanced care for depression across (1) an overall patient population or (2) various high-risk patient groups using structured datasets covering acute and chronic conditions, patient demographics, behaviors, and past visit history. Furthermore, these results show considerable potential to enable preventative care and can be easily integrated into existing clinical workflows to improve access to wraparound health care services.

## Introduction

### Background

Depression is the most commonly occurring mental illness worldwide [1]. It negatively affects how up to 350 million persons worldwide think, feel, and interact [2]. Depression poses significant health and economic burdens to both the individual and community [3]. Previous studies have presented a strong comorbidity between mental health and medical conditions [4]. Depression is highly prevalent among patients suffering from various chronic conditions [5,6]. Such patients may suffer up to a 10-to-25-year reduction in life expectancy [7,8]. Depression is also a leading cause of disability for Americans aged between 15 and 44 years [9]. The incremental economic burden of depression covering medical, pharmaceutical, workplace, and suicide-related costs in the United States was evaluated at US $210.5 billion in 2010, a 21.5% increase from 2005 [10].

Different types of depression pose different levels of risk. Individuals who suffer from mild forms of depression may recover without any assistance. Other less severe cases can be effectively managed by primary care or family practitioners [11-13]. However, other forms of depression are far more severe and require advanced care above and beyond that provided by primary care or family practitioners [14,15]. Identifying cases of depression that require advanced care may be challenging to primary care providers and health care team members whose skill sets run broad rather than deep. Training health care teams to successfully identify patients with severe depression would resolve the problem but is unfeasible given cost, effort, and time considerations [16,17]. Social stigma and ignorance of health issues also encourage depression sufferers to downplay their condition, further increasing difficulty in detection and assessment [18].

Many health care systems leverage screening tools such as the Beck Depression Scale [19], the Patient Health Questionnaire-9 (PHQ-9) [20], PHQ-15 [21], the Cornell Scale for Depression in Dementia [22], and the Hamilton Rating Scale for Depression [23] to evaluate depression severity. However, such tools are not optimal as they (1) tie up significant resources [24], (2) rely heavily on potentially inaccurate patient-reported outcomes for decision making [25], and (3) utilize only a small subset of clinical and behavioral data for decision making. In addition, traditional depression screening approaches may increase risk of overdiagnosis and overtreatment of depression across community and primary care settings [26-28] without contributing to better mental health [29]. Recent studies have questioned the benefits of routine screening [30,31] as well as the US Preventive Services Task Force recommendations to screen adults for depression in primary care settings where staff-assisted depression management programs are available [29].

Given such limitations, it is more appropriate to develop machine learning–based screening approaches capable of leveraging more comprehensive patient datasets representing a patient’s overall health status to identify individuals who cannot be treated at primary care alone and would suffer from worsening health conditions unless they are provided with specialized, high-intensity treatment for depression [14,15]. Machine learning enables us to learn from multiple primary and secondary care datasets that might be missed by a clinician because of cognitive burden, and therefore, are a suitable solution to this challenge.

### Objectives

For purposes of this research, we have defined individuals whose quality of life and health status will degrade if they do not receive specialized treatment above and beyond primary care as patients in need of advanced care for depression. Operationally, such patients would be identified by evaluating clinical data to detect patients who had received referrals to a certified mental health provider for specialized treatment for depression, indicating that their illnesses cannot be treated at primary care alone. In this study, we leveraged data obtained from varied structured and unstructured datasets to build decision models capable of identifying patients in need of advanced care for depression.

## Methods

### Patient Population

We identified a population of 84,317 adult patients (≥18 years of age) with at least 1 primary care visit between the years 2011 and 2016 at Eskenazi Health, a leading health care provider in Indianapolis, Indiana.

### Patient Subset Selection

We sought to predict the need for advanced care for depression across (1) the overall patient population and (2) different groups of high-risk patient populations. We selected 3 high-risk patient groups: group A: patients with a past diagnosis of depression, group B: patients with a Charlson Comorbidity Index [32] of ≥1, and group C: patients with a Charlson Comorbidity Index of ≥2. Patients with a past diagnosis of depression were flagged as a high-risk group as their illness may re-emerge or worsen based on other health conditions. Patients with Charlson indexes ≥1 and ≥2 were selected because of the high prevalence of depression among patients suffering from one or many chronic illnesses [33] and its ability to worsen health outcomes of patients. Thresholds of ≥1 and ≥2 were selected because they captured patient populations that were adequately large for machine learning processes, as well as the cost/effort of potential implementation. We also identified a fourth group (Group D) that comprised all unique patients identified in groups A to C.

We trained models for different populations to capture as many of the overall number of patients in need of advanced care for depression and to identify which patient groups were most suitable for use in screening for need of advanced care. Furthermore, focusing on a smaller population of high-risk patients may be easier to operationalize and cost-efficient to implement across chronic care clinics. Groups A to D were identified by analyzing diagnostic data on each of the 84,317 unique patients (master patient list) for past diagnosis of depression and to calculate Charlson Comorbidity Index for each patient.

### Data Preparation

In a previous effort, we developed a depression taxonomy [34] using knowledge-based terminology extraction of the Unified Medical Language System (UMLS) Metathesaurus [35]. The taxonomy was developed by performing a literature search on Ovid Medline to identify publications that discuss depression and its treatment and then using Metamap [36], a Natural Language Processing–based tool to map these abstracts against the UMLS Metathesaurus, a large, multipurpose, multilingual thesaurus that contains millions of biomedical and health-related concepts, synonymous names, and their relationships across 199 medical dictionaries [37]. The most frequently occurring UMLS concepts were compiled into a terminology using the Web Ontology Language, a semantic Web language that is widely used to represent ontologies. These features presented a wide variety of diagnostic, demographic, and behavioral features that impacted the onset and severity of depression [34].

We obtained longitudinal health records on each patient from the Indiana Network for Patient Care (INPC), a statewide health information exchange [38,39]. Thus, our dataset included records on each patient, including data that may have been captured at any hospital system that participated in the INPC. The dataset included a wide array of patient data, including patient demographic, diagnostic, behavioral, and visit data reported in both structured and unstructured form. All diagnostic data were obtained in the form of structured International Classification of Diseases, ninth revision (ICD-9) and ICD-10 codes. We assessed extracted data against the depression terminology and used relationships presented within the UMLS Metathesaurus to identify ICD codes for inclusion as features. We tabulated vectors of features for each patient group under study. We predict current risk levels based on past patient data. We did not assess the impact of temporality because of our dataset representing a (1) relatively short time period and (2) an older population with high chronic conditions that do not change significantly over time. In the event that the patient under study had received a referral for depression treatment, the data vector only comprised medical data recorded up to 24 hours before the aforesaid referral order. If no past referrals for depression treatment were present, then the vector comprised all available data on the patient. A master data vector encompassing all 84,317 patients was also created using the same approach.

### Preparation of Gold Standard

We applied regular expressions to physician referrals to certified mental health providers to identify referrals where the physician was recommending specialized treatment for depression. We determined that our use of regex patterns was 100% accurate via manual review.

### Decision Model Building

We split each of the 5 data vectors (4 patient subgroups and 1 master data vector) into random groups of 90% training data and 10% test data. Each training dataset was used to train a decision model using the random forest classification algorithm [40]. The random forest algorithm was selected because of its track record of successful use in decision modeling for health care challenges [41,42] and its ability to develop interpretable machine learning predictions [43]. We used Python programming language (version 2.7.6) for all data preprocessing tasks and the Python scikit-learn package for decision model development and testing [44].

### Analysis

Each decision model was evaluated using the 10% holdout test set. Results produced by each decision model were evaluated using area under the curve (AUC) values, which measure classifier accuracy. Youden J Index [45] was used to identify optimal sensitivity and specificity for each decision model.

**Figure 1:**
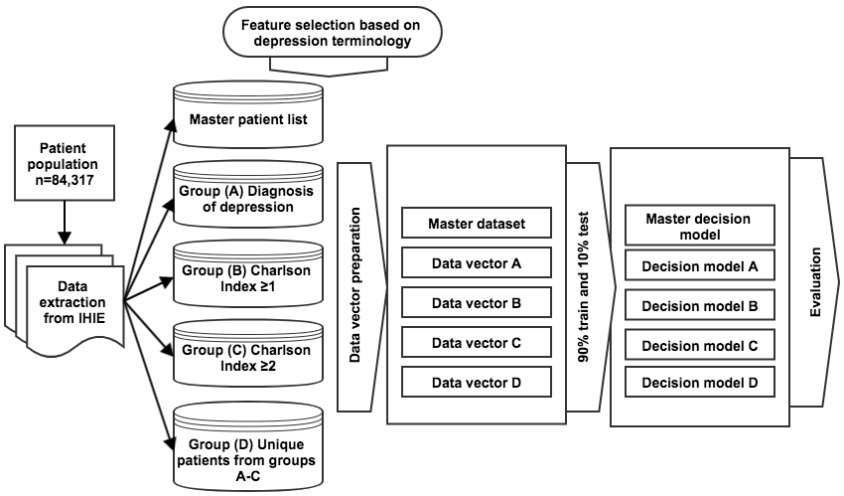
A flowchart representing our workflow from patient group selection to decision model evaluation. IHIE: Indiana Health Information Exchange.

A flowchart representing our workflow from patient group selection to decision model evaluation can be seen in Figure 1.

## Results

### Evaluation of Patient Groups

We identified a total of 12,432 patients with a diagnosis of depression (group A), 32,249 patients with a Charlson Index of 1 or greater (group B), and 7415 patients with a Charlson Index of 2 or more (group C). Overall, these 3 groups identified a total of 37,560 unique patients (group D).

The master patient list as well as each of the 4 high-risk patient groups represented an adult, urban population: predominantly female and with high disease burdens (Table 1). The populations identified by their Charlson Indexes were older (mean age >50 years) than the population identified with depression (46.31 mean age). In addition, populations identified based on Charlson Indexes were predominantly African American. In contrast, the population with a past diagnosis of depression was predominantly composed of non-Hispanic whites. As anticipated, the prevalence of depression across a patient population with a Charlson Index of 1 or greater (30.18%) and a patient population with a Charlson Index of 2 or greater (37.25%) was greater than across the master patient list (19%).

**Figure 2:**
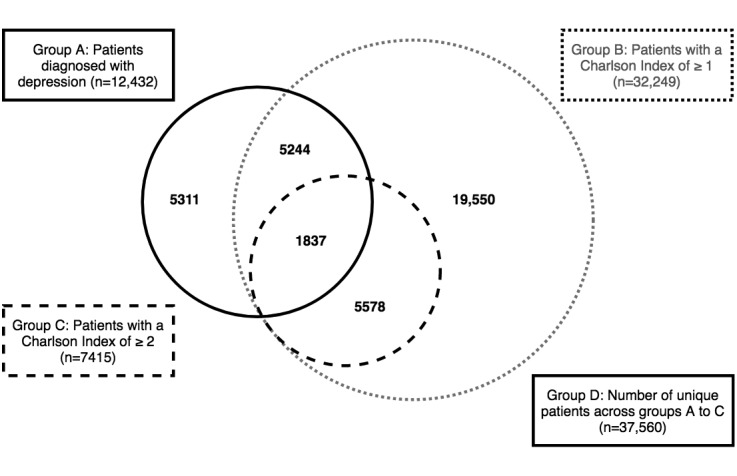
Overlap between the patient groups identified for the study.

Figure 2 presents a Venn diagram presenting overlap across the high-risk patient groups identified for the study.

A total of 6992 (8.29%) of the 84,317 patients in the master patient list were in need of advanced care for depression. Group A captured 3683 (52.68%) of these patients, group B captured 4016 (57.43%), and group C captured 1026 (14.67%). Overall, all 3 patient groups were able to identify 5612 (80.26%) of all patients in need of advanced care for depression.

**Table 1:** Characteristics of the master patient list/groups of high-risk patients used for decision model building.

Characteristic of interest			Master patient set: all patients (N=84,317)	Group A: patients with a past diagnosis of depression	Group B: patients with a Charlson Index of ≥1	Group C: patients with a Charlson Index of ≥2	Group D: all unique patients in groups A-C
Patient group size, n (%)			—^a^	12,432 (14.74)	32,249 (38.25)	7415 (8.8)	37,560 (44.5)
Need of advanced care for depression, n (%)			6992 (8.29)	3683 (30.04)	4016 (12.94)	1026 (21.6)	5612 (80.26)
**Demographics**							
	Age (years), mean (SD)		43.88 (15.60)	46.31 (14.74)	51.94 (14.55)	59.50 (12.33)	50.31 (14.93)
	Male gender (%)		35.09	30.22	39.8	43.98	42.03
	**Race/ethnicity (%)**						
		White (non-Hispanic)	25.21	44.62	33.38	37.02	35.31
		African American (non-Hispanic)	37.23	32.01	42.78	47.26	40.12
		Hispanic or Latino	19.47	11.12	10.60	4.94	7.38
	**Diagnoses**						
		Depression (%)	19.07	100	30.18	37.25	37.51
		Charlson Index score, mean (SD)	0.77 (1.21)	0.22 (0.75)	1.89 (1.27)	3.85 (1.14)	1.62 (1.35)
	**Hospitalizations, mean (SD)**						
		ED^b^ visits during current month	0.21 (1.03)	0.33 (1.48)	0.26 (1.15)	0.31 (1.14)	0.27 (1.17)
		ED visits before previous months	3.73 (14.40)	4.69 (18.73)	8.63 (24.2)	10.71 (31.36)	8.03 (23.67)

^a^Not applicable.

^b^ED: emergency department.

### Feature Selection Using the Depression Terminology

Comparison of patient data against the depression terminology resulted in the identification of 1150 unique concepts for inclusion in each decision model. A description of features included in each of the decision models is presented in Multimedia Appendix 1.

### Decision Model Performance

**Figure 3:**
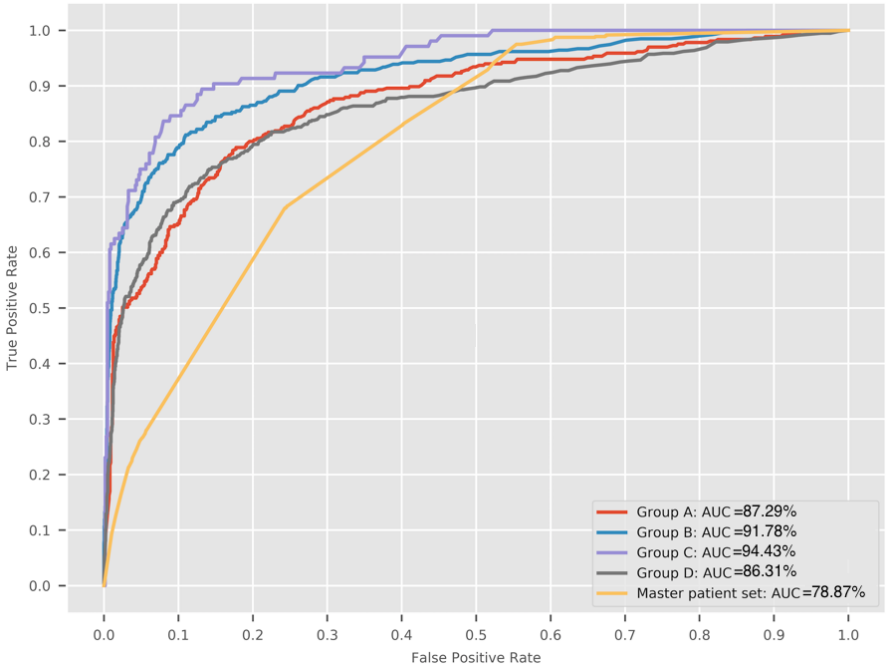
Receiver operating characteristic curves produced by decision models predicting need of advanced care across each patient group under study. AUC: area under the curve.

The decision model predicting need of advanced care across the master population reported a moderate AUC score of 78.87% (optimal sensitivity=68.79%, optimal specificity=76.30%). However, decision models to predict need of advanced care across patients’ groups A to D performed significantly better. Group A (patients with a past diagnosis of depression) reported an AUC score of 87.29% (optimal sensitivity=77.84%, optimal specificity=82.66%). Group B (patients with a Charlson Index of ≥1) reported an AUC score of 91.78% (optimal sensitivity=81.05%, optimal specificity=89.21%). Group C (patients with a Charlson Index of ≥ 2) reported an AUC score of 94.43% (optimal sensitivity=83.91%, optimal specificity=92.18%), whereas Group D (list of unique patients from groups A-C) reported an AUC score of 86.31% (Figure 3; optimal sensitivity=75.31%, optimal specificity=76.03%). Precision-recall curves for each decision model are presented in Multimedia Appendix 2.

The top 20 features for each decision model can be seen in Multimedia Appendix 3. Multimedia Appendix 4 presents the co-occurrence of these top 20 features across each decision model under study. In assessing the top ranked features selected for each decision model, we found significant overlap among the top features for each of the high-risk patient populations. Furthermore, essential (primary) hypertension, depression, gender, and number of outpatient visits appears in the top 20 feature lists for every patient population under test.

To demonstrate that the models did not suffer from overtraining, we added an additional evaluation step where we compared model performance across smaller feature subset sizes. We ranked all features for each decision model using information gain aka. Kullback-Leibler divergence [46]. For each patient subgroup, we used the ranked feature lists to build multiple decision models starting with a decision model trained using only the 5 top ranking features, iteratively adding on the next most important feature, retraining the model and evaluating performance using F1 core. We continued this process until we had trained n-5 models using all n features in the feature set. As an example, for patient group A, we began by building a decision model consisting of 5 patient-centric features and assessing its performance using F1 score. Afterwards, we added in the 6th most important feature and retrained a decision model consisting of these 6 features. We continued building models and evaluating F1 scores until we had included all features from each dataset. The results of this exercise (Multimedia Appendix 5) demonstrated that model performance plateaued after the top 10 to 20 features and that inclusion of further features did not improve model performance. This demonstrates that the models were not overfit and that they reached optimal performance after a relatively small number of features.

## Discussion

### Principal Findings

The decision model to predict need of advanced care for depression across the overall patient population achieved an AUC score of 78.87%. In comparison, decision models that predicted need of advanced care across 4 high-risk patient groups performed better, with AUC scores ranging from 86.31 to 94.43%. In addition, optimal sensitivity and specificity for each decision model was significantly high and demonstrated the models’ potential for practical implementation.

We attribute the comparatively lower performance of the decision model developed using the overall population to the unbalanced nature of the gold standard [47] caused by the relatively low prevalence (8.29%) of patients in need of advanced care and the sparsity of data available for some of the patients in the overall patient population. The high performance of the decision models built using high-risk patient groups could be attributed to the higher prevalence of patients in need of advanced care. Although various publications have presented approaches to address data imbalance [48,49], we did not pursue such as approach as we wished to focus on demonstrating methods that could be replicated across other datasets that may or may not be imbalanced.

In assessing prediction performance, group C (patients with a Charlson Index ≥2) yielded the highest AUC score (97.43%). Groups A (patients with a diagnosis of depression) and B (patients with a Charlson Index ≥1) reported lesser AUC scores. Group C captured the least number of patients in need of advanced care in comparison with groups A and B. However, it is noteworthy that none of the decision models developed using high-risk populations could capture all patients in need of advanced care. Overall, all 3 models could capture only 80.26% of all patients in need of advanced care. The remainder (19.74%) of the patients in need of advanced care did not qualify for any of the three high-risk patient populations. We hypothesize that a share of the missing 19.74% patients would have fallen into 1 of the 3 high-risk patient groups had more comprehensive data been available, and thus, been eligible for detection.

We present a novel application of machine learning to address a question of significant clinical relevance. We demonstrated the ability to predict the need of advanced care for depression across various patient populations with considerable predictive performance. These efforts can easily be integrated into existing hospital workflows [42]. As wraparound services are not delivered by primary care providers [50], the ability to identify and refer patients in need of such services is extremely important [51]. Our efforts yield a highly accurate, automated approach for identifying patients in need of wraparound services for mental health, which is of growing importance to health care organizations and incentivized by changing reimbursement policies. By predicting the need for advanced care across various high-risk populations, we offer potential implementers the option of selecting the best screening approach that meets their needs. Our approach is also well suited to leverage increasing health information technology adoption and interoperability of health care datasets for community-wide health transformations [52,53]. In the field of population health informatics, it enables organizations to leverage widespread acceptance and use of machine learning for cross organizational collaboration and management of various datasets [53] while giving implementers the freedom to select methods best suited for each hospital system. Furthermore, such applications of predictive modeling could support organization-level population health initiatives as risk stratification is fundamental to identifying those patients who are most in need of services to improve health and well-being. In addition, implementing such solutions at primary care ensure that facilitating the entry of all patients into the health care system is more efficient than stand-alone implementations at each chronic care clinic. Thus, our approach presents the ability to effectively identify need of advanced care for depression without risk of overdiagnosis and overtreatment and without the use of manual screening mechanisms.

There is limited knowledge on the best approach to integrate machine learning approaches into existing clinical workflows. As highlighted above, primary care facilities are the point of entry by which a majority of patients suffering from depression seek care [54,55]. However, application of machine learning solutions to screen every primary care visit may be cost-intensive and inefficient for certain clinical practices. Thus, alternate models to evaluate a subset of high-risk patients in need of advanced care for depression would be useful. The 2 potential high-risk patient populations are (1) patients already diagnosed with depression and (2) patients with chronic conditions, who are thus, are at higher risk of suffering from depression [56]. Models built using these subsets may be more practical and result in better machine learning performance than models built using all primary care patients because of variability of underlying data and higher prevalence of outcome of interest, which enables better model training.

We identified several limitations in our study approach. We adopted a binary (present/absent) flag for each feature used to train decision models. We hypothesize that switching to tabulated counts for each feature will increase the granularity of the feature vector, thereby increasing model performance. The patient group used in our study was obtained from the Eskenazi Health system, a safety-net population with significant health burdens. Thus, our models may not generalize to other commercially insured or broader populations. Our diagnostic data were limited to ICD codes. Integrating medications, laboratory, and clinical procedure data may further improve decision model performance. Furthermore, studies present that social determinants of health such as low-educational attainment, poverty, unemployment, and social isolation may have a significant impact on depression and the need for treatment [57,58]. We propose to expand our models using social determinants of health to assess their impact on decision model performance.

We acknowledge that incompleteness of EMR data [59] may impact model performance. Use of claims data may have enabled us to identify a greater number of patients in need of specified treatment into each of our patient subgroups [60]. Furthermore, our outcome of interest are patients in need of advanced care, as identified by primary care providers. Thus, we were unable to account for patients who received advanced care for depression without a past referral. Such patients could have been identified from claims data and used to augment our gold standard.

We selected the random forest classification algorithm for decision-model building based on the need to develop high performance models that were easily interpretable to our clinical audience [42,43]. Other, more advanced decision-modeling approaches such as neural networks [61] have shown potential to improve machine learning performance across various health care challenges. However, neural networks are more complex, cost-intensive, and difficult to interpret [62], making it harder to gain provider acceptance of such models. In addition, it is unclear if they can contribute to our study given the significant performance measures already achieved using random forest models. We recommend that neural networks be considered in a scenario where the sequence or temporality of clinical events is being evaluated, or where the performance of random forest models is unsatisfactory.

In conclusion, these results present considerable potential to enable preventative care and can be potentially integrated into existing clinical workflows to improve access to wraparound health care services.

### Conclusions

Our efforts demonstrate the ability to identify patients in need of advanced care for depression across (1) an overall patient population and (2) various groups of high-risk patients using a wide range of acute and chronic conditions, patient demographics, behaviors, and past visit history. Although all models yielded significant performance accuracy, models focused on high-risk patient populations yielded comparatively better results. Furthermore, our methods present a replicable approach for implementers to adopt based on their own needs and priorities. However, decision model performance may differ based on the availability of patient data at each health care system. These results show considerable potential to enable preventative care and can be easily integrated into existing clinical workflows to improve access to wraparound health care services.

## Appendix

Multimedia Appendix 1 Types of patient data used in decision-model building.

Multimedia Appendix 2 Precision-recall curve for each decision model under study.

Multimedia Appendix 3 List of 20 top features (ranked in order of best to worst) for each decision model, together with their least absolute shrinkage and selection operator scores.

Multimedia Appendix 4 Co-occurrence of top 20 features across each of the patient populations under test (1=most important, 20=least important).

Multimedia Appendix 5 F1 scores for each decision model trained using iteratively increasing feature subset sizes. For clarity, our plot includes only models trained using the top 50 features for each patient subgroup.

## Abbreviations

AUC: area under the curve
ICD: International Classification of Diseases
INPC: Indiana Network for Patient Care
PHQ: Patient Health Questionnaire
UMLS: Unified Medical Language System
