# Supplementary material for: Identification of Patients in Need of Advanced Care for Depression Using Data Extracted From a Statewide Health Information Exchange: A Machine Learning Approach
Source: J Med Internet Res. 2019 Jul 22;21(7):e13809. doi: 10.2196/13809 (PMC6681643; doi:10.2196/13809)
Supplement: Multimedia Appendix 1 [file jmir_v21i7e13809_app1.docx]

## Appendix A. Types of patient data used in decision-model building

| Data type | Description | Representation in machine learning vector |
| --- | --- | --- |
| Age | Patient age | Continuous variable |
| Other demographics (gender, race, race/ethnicity) | Gender, race/ethnicity | Categorical variables |
| Chronic conditions | Arthritis, asthma, coronary artery disease, cardiac arrhythmias, hypertension, hyperlipidemia, stroke, autism spectrum disorder, cancer, chronic kidney disease, chronic obstructive pulmonary disease, dementia, depression, diabetes, hepatitis, HIV AIDS, osteoporosis, schizophrenia | Boolean variables (0= Not recorded, 1=Positive) |
| Acute conditions: | A total of 1,116 different acute diagnoses | Continuous variables (0= Not recorded, or counts for # of occurrences) |
| Charlson index | Charlson comorbidity index score for each patient | Categorical variables |
| Previous visit history | Emergency visits, impatient visits, outpatient visits | Continuous variables for # of occurrences |
