# Supplementary material for: Identification of Patients in Need of Advanced Care for Depression Using Data Extracted From a Statewide Health Information Exchange: A Machine Learning Approach
Source: J Med Internet Res. 2019 Jul 22;21(7):e13809. doi: 10.2196/13809 (PMC6681643; doi:10.2196/13809)
Supplement: Multimedia Appendix 2 [file jmir_v21i7e13809_app2.docx]

## Appendix B. Precision-Recall curve for each decision model under study


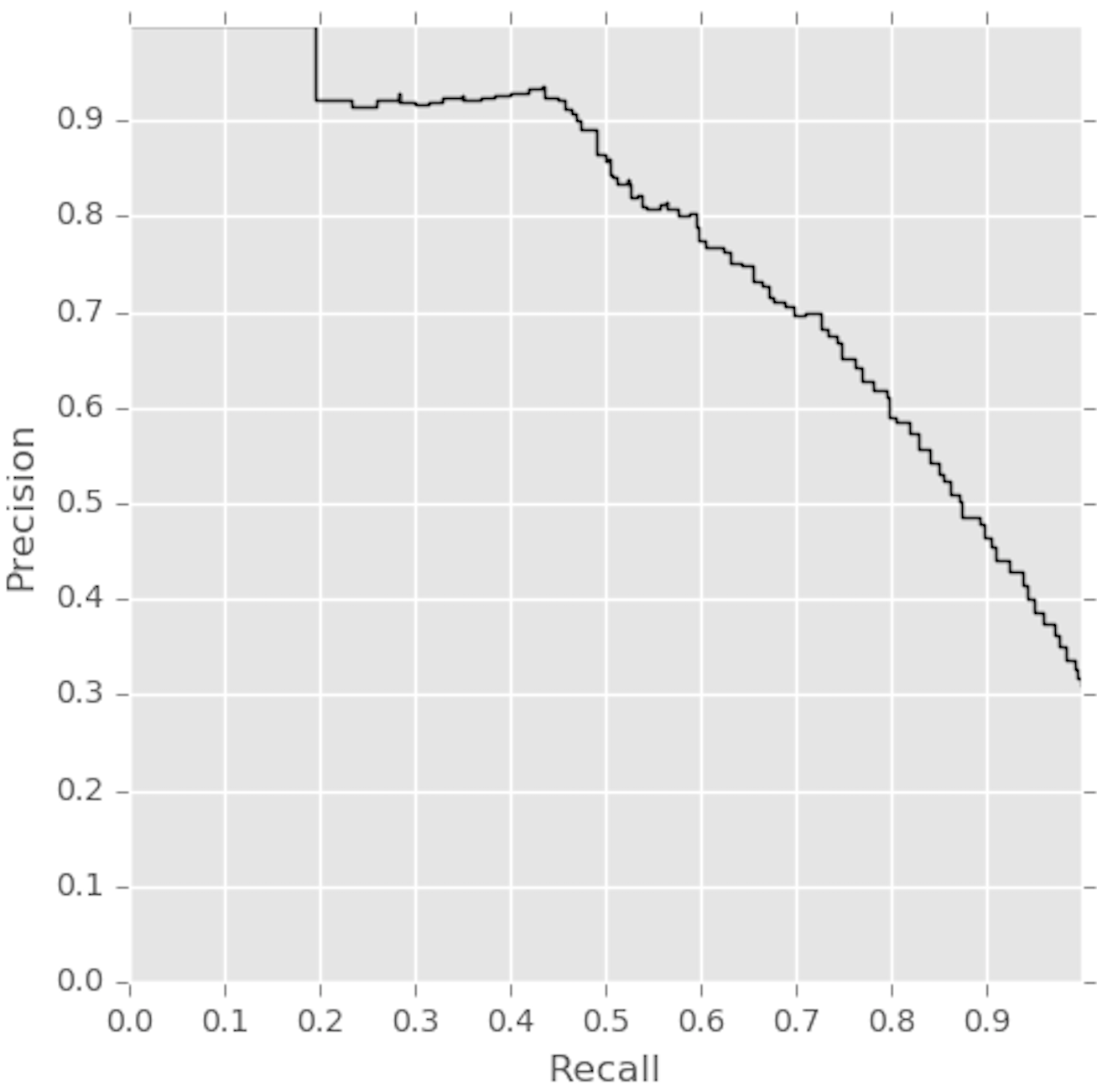


Patients with a prior diagnosis of depression (Group A)


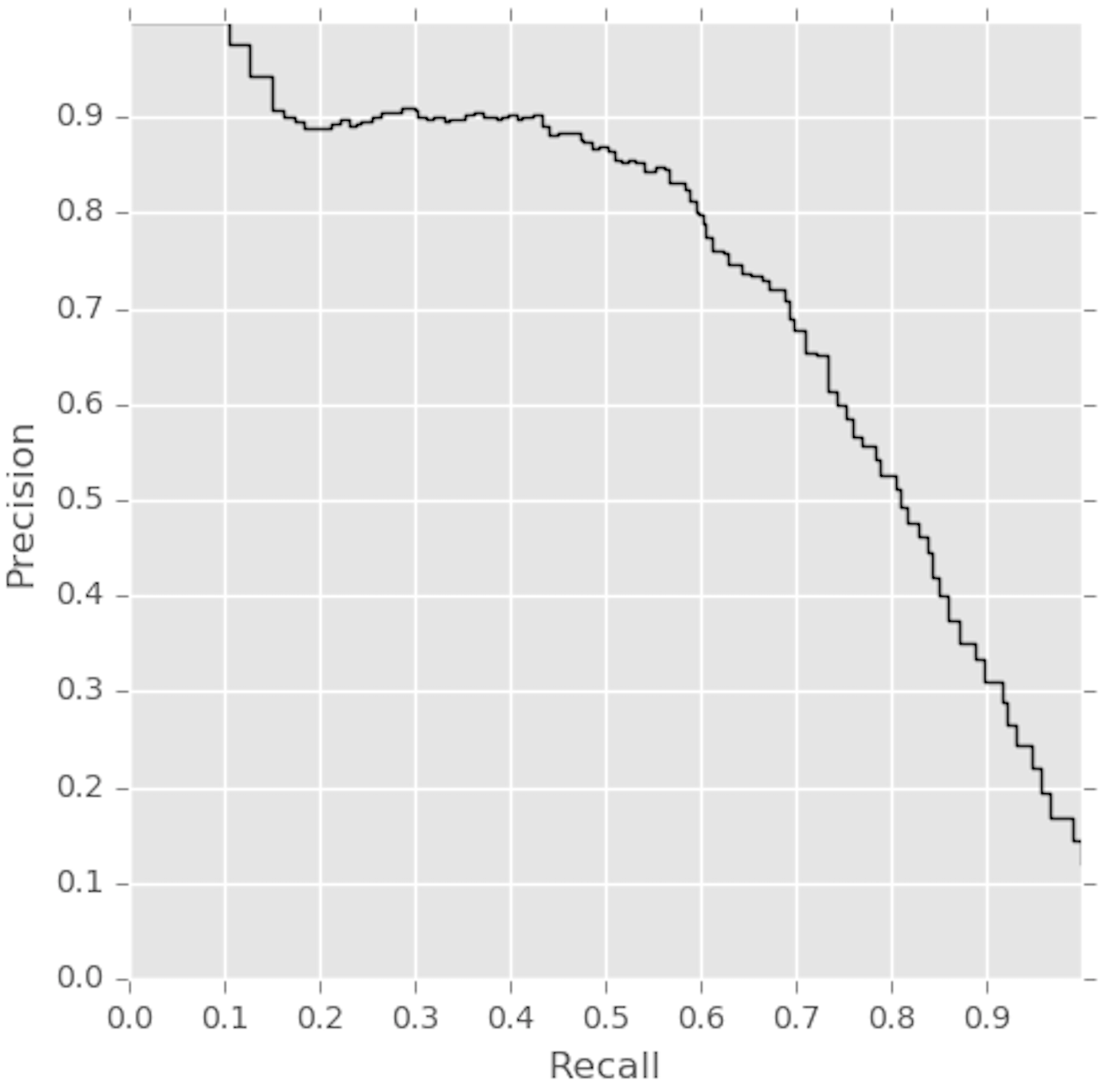


Patients with a Charlson Index of <=1 (Group B)


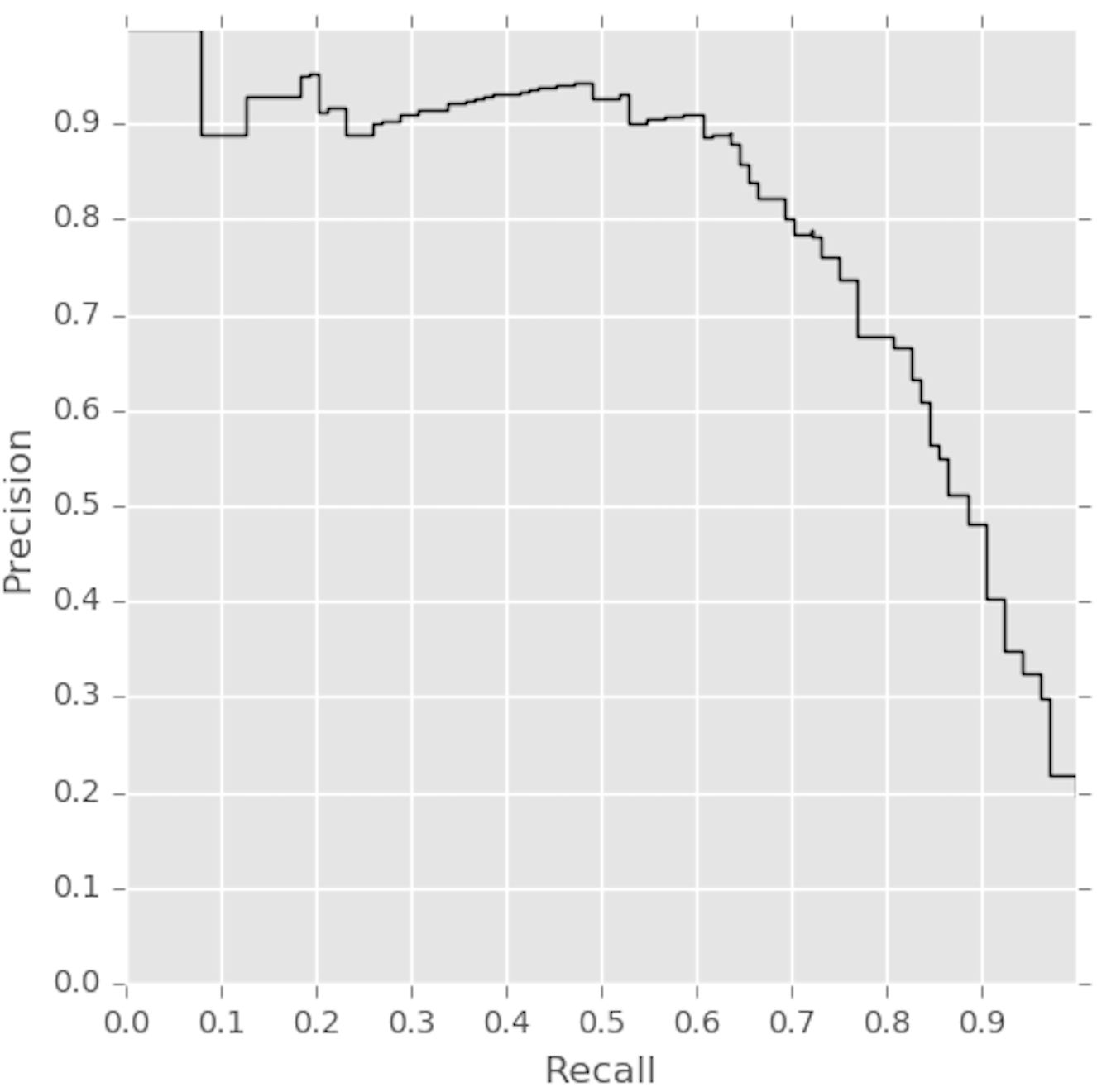


Patients with a Charlson Index of <=2 (Group C)


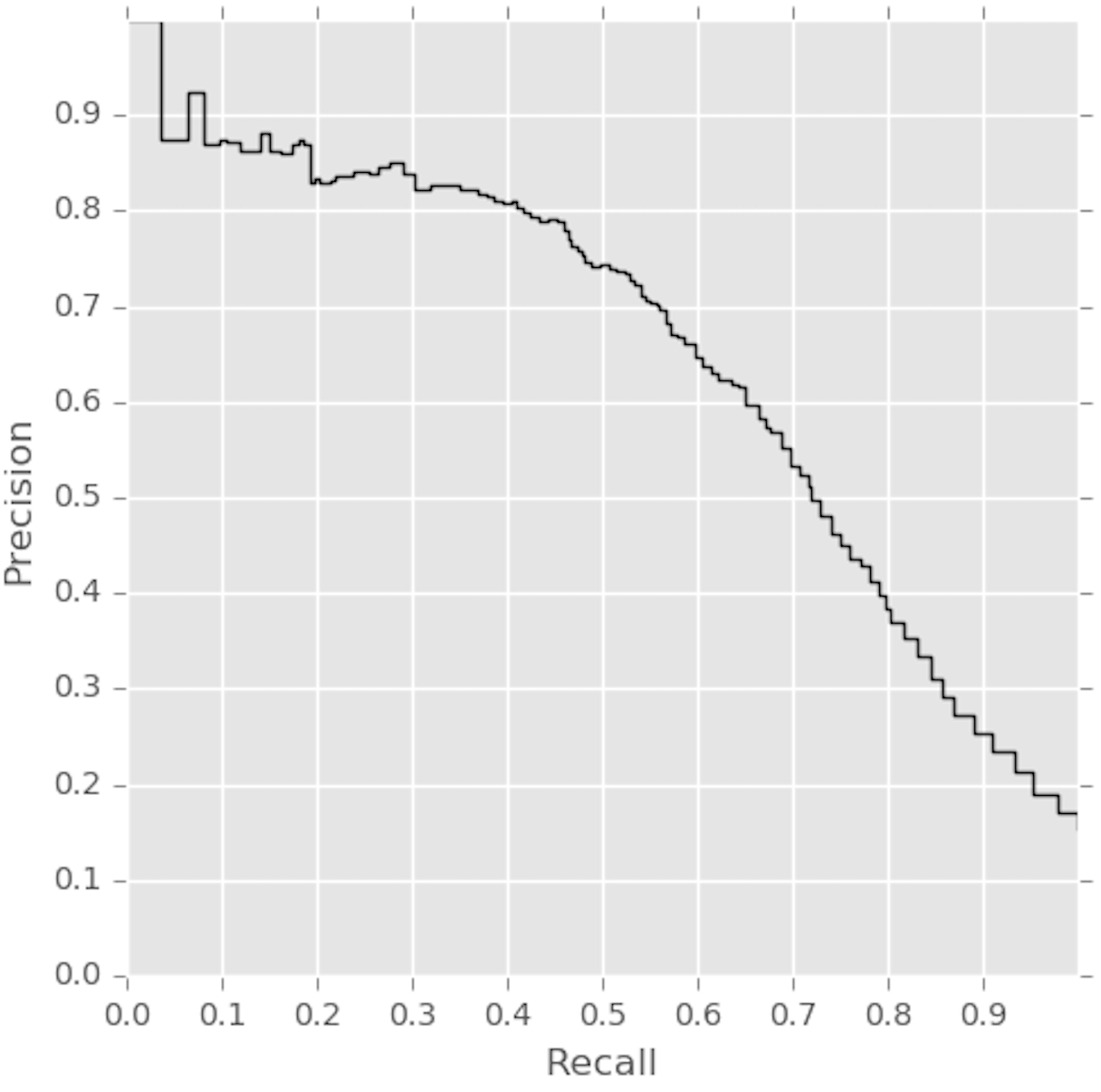


All unique patients in groups A-C (Group D)
