# Supplementary material for: Identification of Patients in Need of Advanced Care for Depression Using Data Extracted From a Statewide Health Information Exchange: A Machine Learning Approach
Source: J Med Internet Res. 2019 Jul 22;21(7):e13809. doi: 10.2196/13809 (PMC6681643; doi:10.2196/13809)
Supplement: Multimedia Appendix 3 [file jmir_v21i7e13809_app3.docx]

## Appendix C. List of 20 top features (Ranked in order of best to worst) for each decision model, together with their LASSO scores

| # | Master patient population | Group A | Group B | Group C | Group D |
| --- | --- | --- | --- | --- | --- |
| 1 | Essential (primary) hypertension  (1.0) | Number of outpatient visits > last 30 days  (1.0) | Number of outpatient visits > last 30 days (1.0) | Number of outpatient visits > last 30 days (1.0) | Number of outpatient visits > 30 days (1.0) |
| 2 | Depressive disorder (0.765) | Schizophrenia (1.0) | Depressive disorder (1.0) | Number of inpatient visits prior to last 30 days (1.0) | Depressive disorder (1.0) |
| 3 | Dorsalgia  (0.5) | Hyperlipidemia (1.0) | Charlson Index (1.0) | Number of inpatient visits during last 30 days (1.0) | Gender (1.0) |
| 4 | Nicotine dependence  (0.5) | External injury (1.0) | Gender (1.0) | Hyperlipidemia (1.0) | Essential (primary) hypertension  (1.0) |
| 5 | Joint disorder  (0.48) | Depressive disorder (1.0) | Essential (primary) hypertension (1.0) | External injury (1.0) | Diabetes mellitus  (1.0) |
| 6 | Gender (0.48) | Arthritis (1.0) | Episodic mood disorders (1.0) | Depressive disorder (1.0) | Charlson Index (1.0) |
| 7 | Encounter for contraceptive management (0.475) | Nonspecific findings on examination of blood  (1.0) | Diabetes mellitus (1.0) | Charlson Index (1.0) | Asthma (1.0) |
| 8 | Routine general medical examination  (0.475) | Other cerebral degenerations  (1.0) | Hyperlipidemia (0.87) | Arthritis (1.0) | Disorders of lipoid metabolism  (0.95) |
| 9 | Examination of eyes and vision  (0.465) | Cancer (0.995) | Cancer (0.77) | Age (1.0) | Other cerebral degenerations  (0.9) |
| 10 | Abdominal and pelvic pain  (0.465) | Vitamin D deficiency  (0.995) | Other cerebral degenerations  (0.715) | Gender (1.0) | Symptoms of the respiratory system and other chest symptoms  (0.85) |
| 11 | Encounter for screening for malignant neoplasms  (0.46) | Charlson Index (0.985) | Asthma (0.6) | Chronic kidney disease (1.0) | Bronchitis (0.8) |
| 12 | Soft tissue disorders  (0.46) | Episodic mood disorders  (0.805) | Number of inpatient visits prior to last 30 days (0.555) | Chronic airway obstruction (1.0) | Number of emergency department visits prior to last 30 days (0.77) |
| 13 | Long-term (current) drug therapy (0.455) | Cataract (0.8) | Bronchitis (0.555) | Other and ill-defined cerebrovascular disease (1.0) | Number of inpatient visits prior to last 30 days (0.723) |
| 14 | Number of outpatient visits prior to last 30 days (0.43) | Asthma (0.59) | External injury (0.55) | Heart failure (1.0) | Hyperlipidemia (0.66) |
| 15 | Episodic mood disorders (0.22) | Heart failure (0.545) | Arthritis (0.545) | Acute myocardial infarction (0.995) | Disorders of fluid electrolyte and acid-base balance  (0.635) |
| 16 | Pain in throat and chest  (0.175) | Number of emergency department visits during last 30 days (0.54) | Disorders of fluid electrolyte and acid-base balance (0.515) | Essential (primary) hypertension (0.95) | Persistent mental disorders  (0.6) |
| 17 | Encounter for screening for infectious and parasitic diseases  (0.175) | Chronic kidney disease  (0.525) | Nondependent abuse of drugs  (0.51) | Asthma (0.925) | Chronic obstructive pulmonary disease (0.612) |
| 18 | Encounter for other special examination without complaint  (0.15) | Sprain of neck  (0.52) | Number of inpatient visits during last 30 days (0.49) | Nondependent abuse of drugs  (0.9) | Arthritis (0.6) |
| 19 | Anxiety, dissociative and somatoform disorders  (0.125) | Gender (0.515) | Number of emergency department visits prior to last 30 days (0.49) | Diabetes mellitus  (0.85) | Acute myocardial infarction  (0.55) |
| 20 | Diabetes mellitus  (0.05) | Attention deficit disorder without mention of hyperactivity  (0.515) | Persistent mental disorders  (0.48) | Other peripheral vascular disease  (0.85) | Dementia (0.51) |
