# Supplementary material for: Identification of Patients in Need of Advanced Care for Depression Using Data Extracted From a Statewide Health Information Exchange: A Machine Learning Approach
Source: J Med Internet Res. 2019 Jul 22;21(7):e13809. doi: 10.2196/13809 (PMC6681643; doi:10.2196/13809)
Supplement: Multimedia Appendix 4 [file jmir_v21i7e13809_app4.docx]

## Appendix D. Co-occurrence of top 20 features across each of the patient populations under test (1 = most important, 20 == least important)

| Feature | Master patient population | Group A | Group B | Group C | Group D |
| --- | --- | --- | --- | --- | --- |
| Essential (primary) hypertension | 1 |  | 5 | 16 | 4 |
| Depressive disorder | 2 | 5 | 2 | 6 | 2 |
| Dorsalgia | 3 |  |  |  |  |
| Nicotine dependence | 4 |  |  |  |  |
| Joint disorder | 5 |  |  |  |  |
| Gender | 6 | 19 | 4 | 10 | 3 |
| Encounter for contraceptive management | 7 |  |  |  |  |
| Routine general medical examination | 8 |  |  |  |  |
| Examination of eyes and vision | 9 |  |  |  |  |
| Abdominal and pelvic pain | 10 |  |  |  |  |
| Encounter for screening for malignant neoplasms | 11 |  |  |  |  |
| Soft tissue disorders | 12 |  |  |  |  |
| Long-term (current) drug therapy | 13 |  |  |  |  |
| Number of outpatient visits prior to last 30 days | 14 | 1 | 1 | 1 | 1 |
| Episodic mood disorders | 15 | 12 | 6 |  |  |
| Pain in throat and chest | 16 |  |  |  |  |
| Encounter for screening for infectious and parasitic diseases | 17 |  |  |  |  |
| Encounter for other special examination without complaint | 18 |  |  |  |  |
| Anxiety, dissociative, and somatoform disorders | 19 |  |  |  |  |
| Diabetes mellitus | 20 |  | 7 | 19 | 5 |
| Schizophrenia |  | 2 |  |  |  |
| Hyperlipidemia |  | 3 | 8 | 4 | 14 |
| External injury |  | 4 | 14 | 5 |  |
| Arthritis |  | 6 | 15 | 8 | 18 |
| Nonspecific findings on examination of blood |  | 7 |  |  |  |
| Other cerebral degenerations |  | 8 | 10 |  | 9 |
| Cancer |  | 9 | 9 |  |  |
| Vitamin D deficiency |  | 10 |  |  |  |
| Charlson Index |  | 11 | 3 | 7 | 6 |
| Cataract |  | 13 |  |  |  |
| Asthma |  | 14 | 11 | 17 | 7 |
| Heart failure |  | 15 |  | 14 |  |
| Number of emergency department visits during last 30 days |  | 16 |  |  |  |
| Chronic kidney disease |  | 17 |  | 11 |  |
| Sprain of neck |  | 18 |  |  |  |
| Attention deficit disorder without mention of hyperactivity |  | 20 |  |  |  |
| Number of inpatient visits prior to last 30 days |  |  | 12 | 2 | 13 |
| Bronchitis |  |  | 13 |  | 11 |
| Disorders of fluid electrolyte and acid-base balance |  |  | 16 |  | 15 |
| Nondependent abuse of drugs |  |  | 17 | 18 |  |
| Number of inpatient visits during last 30 days |  |  | 18 | 3 |  |
| Number of emergency department visits prior last 30 days |  |  | 19 |  | 12 |
| Persistent mental disorders |  |  | 20 |  | 16 |
| Age |  |  |  | 9 |  |
| Chronic airway obstruction |  |  |  | 12 |  |
| Other and ill-defined cerebrovascular disease |  |  |  | 13 |  |
| Acute myocardial infarction |  |  |  | 15 | 19 |
| Other peripheral vascular disease |  |  |  | 20 |  |
| Disorders of lipoid metabolism |  |  |  |  | 8 |
| Symptoms involving respiratory system and other chest symptoms |  |  |  |  | 10 |
| Chronic obstructive pulmonary disease |  |  |  |  | 17 |
| Dementia |  |  |  |  | 20 |
