# Supplementary figures and images for: Identification of Patients in Need of Advanced Care for Depression Using Data Extracted From a Statewide Health Information Exchange: A Machine Learning Approach
Source: J Med Internet Res. 2019 Jul 22;21(7):e13809. doi: 10.2196/13809 (PMC6681643; doi:10.2196/13809)

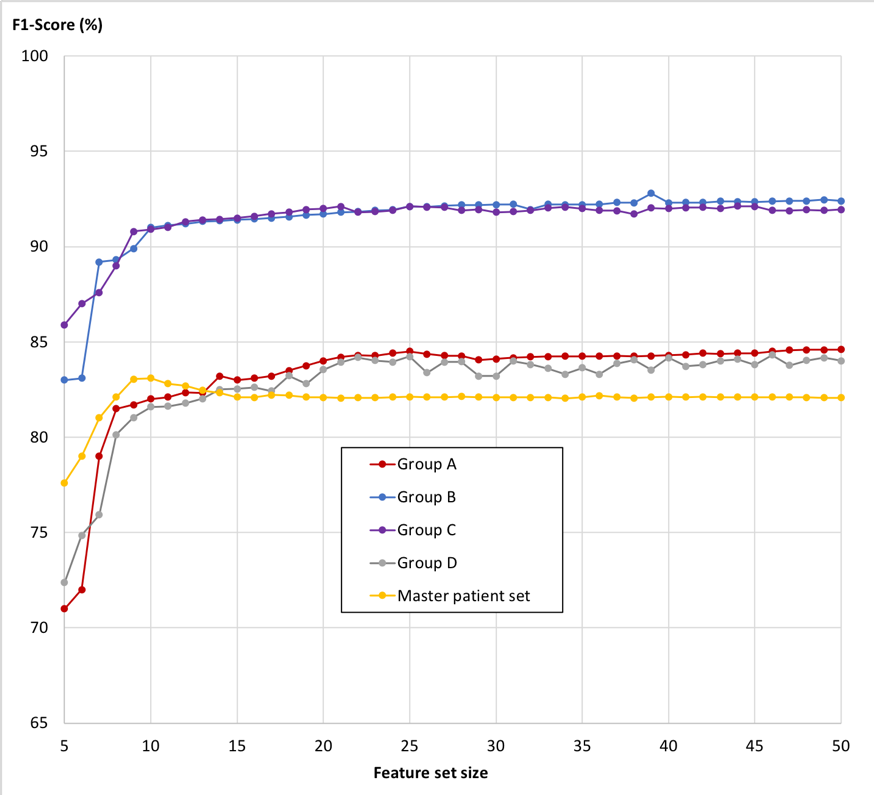

Supplement: Multimedia Appendix 5 [file jmir_v21i7e13809_app5.png]
